# Long-Term Effects of Tai Chi Intervention on Sleep and Mental Health of Female Individuals With Dependence on Amphetamine-Type Stimulants

**DOI:** 10.3389/fpsyg.2018.01476

**Published:** 2018-08-20

**Authors:** Dong Zhu, Guobin Dai, Ding Xu, Xin Xu, Jingjing Geng, Weimo Zhu, Xi Jiang, Marc Theeboom

**Affiliations:** ^1^School of International Education and College of Wushu, Shanghai University of Sport, Shanghai, China; ^2^College of Wushu, Shanghai University of Sport, Shanghai, China; ^3^Health and Rehabilitation Department, Shanghai Drug Administration, Shanghai, China; ^4^College of Kinesiology, Shanghai University of Sport, Shanghai, China; ^5^Kinesiology & Community Health, University of Illinois at Urbana-Champaign, Champaign, IL, United States; ^6^Sports Law Center, Shanghai University of Political Science and Law, Shanghai, China; ^7^Department of Movement and Sport Sciences, Faculty of Physical Education and Physiotherapy, Vrije Universiteit Brussel, Brussels, Belgium

**Keywords:** tai chi, sleep quality, depression, fitness, amphetamine-type stimulants, relapse, women

## Abstract

Previous studies provide evidence that Tai Chi (TC) can reduce the symptoms of sleep problems and be of benefit for the rehabilitation of substance abusers. In this study, we investigated if TC practice can improve sleep quality and mood of females who are dependent on amphetamine-type stimulant (ATS). Eighty subjects were randomly assigned to TC intervention and standard care (SC) for 6 months. We applied analysis of variance on repeated-measure with the year of drug dependence as the covariate to test the changes of the self-rated Pittsburg Sleep Quality Index (PSQI), Self-Rating Depression Scale (SDS), as well as fitness after 3 and 6 months. Relapse investigation was conducted by checking the database of China's National Surveillance System on Drug Abuse and that of the Shanghai Drug Control Committee's illicit drug dependents. Our investigation focused on the relapse of participants who had undergone and completed treatment in the Shanghai Mandatory Detoxification and Rehabilitation Center in 2015. The result showed that the PSQI scores of sleep duration [*F*_(2, 92)_ = 9.86], need for sleep medications [*F*_(2, 92)_ = 36.44] and daytime dysfunction [*F*_(2, 92)_ = 5.15] were found to have a significant difference by time × group interaction after 6 months. SDS showed no significant difference between the two groups; however, the score of SDS in TC decreased after 6-month intervention, and no changes were observed in SC. Pulse rate had significantly decreased in the TC group compared with the SC group after 6 months. 9.5% (4) ATS dependents in TC and 26.3% (10) ATS dependents in SC were found to have relapsed. Our result suggested that TC had positive effects on sleep quality, depression and fitness. Long-term study demonstrated that TC may be a cheap and potential supplementary treatment for ATS-dependent individuals. TC may also be considered as an alternative exercise to escalate abstinence for ATS-dependent females.

Clinical trial registration: ChiCTR-IPR-14005343 chictr. org.cn

## Introduction

Substance abuse is a major public health concern worldwide. Amphetamine-type stimulants (ATSs) are the second most prevalent drugs used globally. ATS use has been correlated with serious health consequences, such as cognitive impairment, poor physical status, high risk of mortality, and increased social burden (Chen et al., 2015). Recently, the population of ATS use in China has grown substantially (Du et al., 2014). The registered drug use identified as methamphetamine (MA) user increased from 29 to 43.8% in 2014 (Zhu et al., 2016). With East Asia regarded as a major region of concern due to its high MA production and trafficking (Rawson, 2013; Gowing et al., 2015; Kittirattanapaiboon et al., 2017), many researchers have raised an alert about ATS use/abuse around the world. The challenges of ATS use disorder have been recognized in China, and a large body of research on ATS use has emerged (Du et al., 2014).

Chronic substance use disorder (SUD) may exhibit symptoms that can include significant insomnia and mood disturbances (Bao et al., 2013). Numerous studies have shown that sleep is disrupted during active use of MA, withdrawal, and abstinence (Lipinska et al., 2015). A cross-sectional study found that the prevalence of sleep disturbance was high in drug users, which was twice the sleep latency of non-drug users. The study further indicated a link between sleep problems and duration of drug use (Tang et al., 2015). Excessive daytime sleepiness in stimulant users, which can result from poor sleep quality and/or reduced sleep time, may lead to partial sleep deprivation and deleteriously affect cognitive functioning. A study revealed that MA use disorder had elevated daytime sleepiness and a significantly higher score of Pittsburgh Sleep Quality Index (PSQI) compared with those with normal daytime sleepiness (Mahoney et al., 2014). However, a report revealed no association between sleep quality and substance use and further indicated that worse sleep was associated with worse mood (Putnins et al., 2012). Depression is another prevalent psychiatric symptom among ATS-dependent individuals. Evidence provided a result to support the consequence that more than half of the ATS dependents had depression symptoms (Du et al., 2014). Sleep quality and mood may play a role in the course of ATS use disorders and affect the outcome of treatment (Putnins et al., 2012).

Men and women abuse the same drugs, but not always in the same ways. Significant gender differences have been reported in the initiation of drug use, reasons for continuing to use drugs and the resumption of drug taking after periods of abstinence (relapse) (Fattore et al., 2014). Women affected by drug dependence and HIV are more vulnerable and more stigmatized than men. They suffer from co-occurring mental health disorders to a greater extent than men, and they are more likely to have been victims of violence and abuse. Women often also bear a heavy burden of violence and deprivation associated with the drug dependence of family members, hindering the achievement of the sustainable development target of eliminating all forms of violence against all women and girls (Greenfield et al., 2007; UNODC World Drug Report, 2016). They need help to build supportive social networks and establish a safe and predictable family environment for themselves and their children (Wiig et al., 2017). Female SUDs encounter significant systemic, structural, social, cultural, and personal barriers in accessing substance abuse treatment (United Kingdom., 2010; Guerrero et al., 2014). Although studies found that sleep duration is longer and sleep quality is higher on average in women than men (Knutson, 2013), women are diagnosed with depression and anxiety disorders roughly twice as often as men (Fattore et al., 2014). Current studies focus on specific vulnerable populations, such as sex workers and women with HIV (Rawson, 2013; Rodriguez et al., 2014; Zhang et al., 2015; Page et al., 2016; Kittirattanapaiboon et al., 2017). However, reports on the treatment of female ATS-dependent individuals are rare.

The main therapy for ATS dependence has focused on the symptomatic treatment related to ATS use complications of acute intoxication, symptoms of withdrawal such as anxiety and depression and mental disorders such as hallucinations and delusions due to chronic ATS abuse (Sun et al., 2014). Physical exercise has long been considered important in preventing and treating several medical conditions (Scully, 1998). Some evidences suggest that exercise can attenuate MA use (Miller et al., 2012) and ameliorate symptoms of depression and anxiety, which are commonly reported among MA users attempting to abstain from drug use and may be associated with drug use relapse (Dolezal et al., 2013; Segat et al., 2014). Physical exercise has been suggested to slow down the decline in cognitive function (Wayne et al., 2014; Zhang et al., 2014). Increased daytime physical activities have also been recommended to improve the quality of nighttime sleep. Exercise may effectively maintain cognitive function and improve the nighttime sleep of elderly people (Chan et al., 2016). Recent studies have focused on alternative forms of physical activity, such as tai chi (TC), which is a traditional Chinese martial art. TC has been described as a mind–body exercise regimen that benefits fitness, muscle strength, flexibility, postural control, and fall-risk reduction, as well as quality of life and well-being (Li et al., 2005; Huang et al., 2017). Several studies have evaluated the effect of TC on psychological responses, including depression, distress, well-being, life satisfaction, and perception of health (Wang et al., 2004; Taylor-Piliae et al., 2006; Garber et al., 2011).

To date, few studies focus on the effect of TC intervention on participants' sleep quality. TC can be considered a useful non-pharmacologic approach to improve sleep quality in older adults with moderate complaints and has the potential to ameliorate sleep complaints possibly before syndromal insomnia develops (Irwin et al., 2008). One study that systematically reviewed studies published between 2004 and 2014 found that TC was a common complementary exercise, which is implemented in sleep quality intervention. In the review, the most common duration of TC intervention was 60 min and the common length was 12 weeks (Wang et al., 2016). Recent studies investigated the effect of TC on heroin and ATS dependents, with reports that TC participants had better psychological and physical outcomes compared with control groups (Li et al., 2013). However, whether TC intervention works on the physical and mental effect of female ATS dependencies is not clear. As TC includes training in sustained attention focusing and multitasking (Fong et al., 2014), the meditation component may have direct benefits by enhancing attention and executive functions.

It is notable that gender differences emerged with regard to the types of exercise as well as the perceived benefits of engaging in exercise-based intervention, Yoga, stretching, and use of exercise videos are more appealing to women (Abrantes et al., 2011). As such, TC may be an effective intervention to influence female ATS dependents mentally and physically. The objective of this study is to assess the effect of TC intervention on female ATS dependents for sleep quality and fitness change at Shanghai Mandatory Detoxification and Rehabilitation Center (SMDRC). We hypothesized that TC intervention may improve sleep quality and mental health for female individuals of ATS dependent.

## Materials and methods

### Design, setting, and participants

This single-blind (assessors-blind), two-group randomized controlled trial was conducted between May 2014 and December 2014. In accordance with the “Narcotic Control Act”, newly found drug users are sent to the drug abuse treatment hospital to assess the severity of their drug use and then back to the community to receive detoxification treatment under the supervision of social workers. If they relapse, drug users are sent to the mandatory detoxification and rehabilitation center for drug rehabilitation, where they participate in a combination of detoxification treatment, physical exercise and manual labor for 2 years (Du et al., 2014). Relapse investigation was conducted through a data check with China's National Surveillance System on Drug Abuse (NSSDA) and using the database of illicit drug dependents monitored by the Shanghai Drug Control Committee (SDCC). The target group monitored by NSSDA consisted of exposed illicit drug-dependent individuals. The monitoring coverage by this database in China is 96.3% (Cong et al., 2013). The database of SDCC lists illicit drug-dependent individuals who ever had or are receiving rehabilitation at SMDRC. Illicit drug dependents who have left from SMDRC are monitored by these systems.

The participants were all female ATS-dependent individuals. At the time of recruitment, these individuals were receiving drug withdrawal treatment at a female SMDRC. The inclusion criteria comprised voluntary individuals who were (1) aged 18 years or above, (2) level 3 illicit drug-dependent users (assessed by using the Chinese version of Addiction Severity Index and classified in accordance with the “Regulations on Prohibition against Narcotics”) (Li et al., 2010) and (3) ATS dependents identified by the Guidelines of Diagnostic and Treatment on ATS Use issued by the Chinese Ministry of Health (Sun et al., 2014) and (4) had no severe medical conditions that would preclude their participation in physical activities. The exclusion criteria consisted of (1) diagnosis of Axis I psychiatric disorders in addition to SUD, (2) medical or neurological illnesses or trauma that affects the central nervous system, and (3) undergoing pharmacological treatment with psychotropic medications.

### Intervention

The participants were all female and were randomly assigned by computer-generated random numbers to either the TC (*n* = 40) or standard care (SC) groups (*n* = 40). As two participants were reallocated in accordance with the duration of their stay in SMDRC, the final numbers of participants were 42 in TC and 38 in SC. The study protocol was approved by the ethical committees of the Shanghai University of Sport and SDCC. They participated in exercise sessions five times a week during the first 3 months and three times a week during the second 3 months. These interventions conducted in SMDRC are described subsequently.

#### TC group

The exercise taught to the TC group was based on a simplified 24-form TC. Movements were modified in accordance with the physical capabilities and psychological characteristics of the ATS dependents, which emphasized multidirectional weight-shifting, awareness of body alignment and multisegmental (arms, legs, and trunk) movement coordination (Li et al., 2004). Each session approximately consisted of a 10 min warm-up, 40 min TC exercise and 10 min cooldown. The objective was to provide a safe exercise that illicit drug dependents in SMDRC can participate in as a supplementary treatment. TC is one of the recommended exercises by SNCC. The movements and intensity of modified TC have been described in detail by Zhu et al. in our previous study (Zhu et al., 2016). One professional TC instructor from the Shanghai University of Sport instructed and supervised the TC group.

#### SC group

The exercises in the SC group had similar exercise intensity as the experimental group, which included a 5 min recreation activity (Guang Bo Ti Cao), 5 min gesture language exercises and a self-study as recommended by SNCC. The SC model is widely applied among mandatory detoxification and rehabilitation centers in Shanghai. The ninth edition of Guang Bo Ti Cao was designed by the China General Administration of Sports. This exercise is divided into eight sections and lasts for 4 min and 45 s. The gesture language exercise consists of an upper limb exercise. Qualified instructors from the SMDRC instructed the SC group. Self-education, which was performed after the exercises, included reading books related to the knowledge of health and watching TV. The duration of the SC intervention was similar to that of the TC intervention.

### Procedure

The study was performed in a female SMDRC. The participants were informed of the purpose of this study and were asked to sign a consent form during the admission process. The study was performed in accordance with the Declaration of Helsinki II. TC and SC interventions were performed on a basketball field under fair weather or in an indoor self-education room during rainy weather. The intervention outcomes were assessed at the baseline and after 3 and 6 months. Experienced researchers conducted the assessment and were blinded to the two groups. A follow-up relapse investigation was conducted.

### Outcome measures

Outcome measures were obtained at the baseline and after 3 and 6 months, to verify the changes in sleep quality, depression, and physical effects among the subjects of the two groups as a result of the interventions.

#### Self-rated sleep quality

The self-rated sleep quality was measured by using the PSQI to evaluate the quality and pattern of sleep at the baseline and after 3 and 6 months, for ATS dependents. The PSQI, with 19 items, is a widely used questionnaire to generate seven sleep component scores, including subjective sleep quality, sleep latency, sleep duration, habitual sleep efficiency, sleep disturbances, need for sleep medications, and daytime dysfunction. The sum of these component scores yields a global score (range: 0–21), with a score cutoff of greater than 5 indicating a clinical sleep impairment with a high intensity and specificity in identifying insomnia (Li et al., 2004; Peles et al., 2006; Irwin et al., 2008).

#### Self-rating depression scale (SDS)

The SDS developed by Zung was used to measure the depression level during 6 months of intervention. The advantages of this scale include ease of use in self-rating and its applicability to a wide range of people, from healthy individuals to patients (Baba et al., 2015). The SDS is a 20-item self-assessment questionnaire that measures depression. Item scores range from 1 to 4 (total score range: 20–80) (Jegede, 1976; Sashika et al., 2017). The raw scores are cataloged into four levels: 20–39 (normal range), 40–47 (mildly depressed), 48–55 (moderately depressed), and 56 and above (severely depressed) (Yang et al., 2016).

#### Fitness evaluation

The fitness tests were administrated by experienced investigators. Measurements at the baseline and after 3 and 6 months were performed in the morning at the same time. Blood pressure was measured under standardized conditions prior to other tests: participants were asked to rest for 5 min and had not taken any caffeine or tobacco products within 30 min. Body composition and body mass index were measured with Omron HBF-305. Hand grip power test was assessed using a grip dynamometer, and flexibility was tested with a sit and reach test equipment. Balance test was performed with one leg stand with eyes closed. A sport watch was used to record the duration of one leg stand with eyes closed. The progressive aerobic cardiovascular endurance run (PACER) was performed to measure the aerobic capacity of ATS dependents with following standardized procedures. The participants ran from one marker to another marker set 20 m apart while keeping pace with a pre-recorded cadence. The cadence was set to music and increased every minute. Participants were instructed to keep up with the cadence for as long as possible. The test was terminated when a participant failed to reach the appropriate marker in the allotted time twice or could no longer maintain the pace. The number of laps completed was recorded (Mahar et al., 2011; Marques et al., 2015).

### Relapse investigation

NSSDA is part of law enforcement and managed administratively by judicial departments or public security bureaus. The sites monitor the prevalence of drug users (both relapsed and new) and types of drugs used and facilitate detoxification treatment for drug users. The information collected includes basic identification and demographic information, drug type, first time drug use, results of urine tests, main places of drug use and any disease comorbidity (Cong et al., 2013; Jia et al., 2015). The ATS-dependent individual relapse in this study was investigated on the basis of NSSDA data and combined with the latest records administrated by SDCC. The content of the relapse investigation was based on data including the name and exact date of relapse for participants who had taken part in this experiment. ATS participants received treatment from July 2012 to July 2013 and were discharged to the community from July 2014 to July 2015 after 2 years of mandatory treatment in this study. The relapse investigation was conducted on June 25, 2017, to examine who has relapsed after they took part in this study.

### Statistics

Statistical analyses were performed using SPSS 19.0 (Chicago, USA). Pearson chi-squared test was applied for categorical variables of demography, and independent sample *t*-test was applied for continuous variables at the baseline comparison of PSQI, SDS, fitness and relapse. Pearson chi-squared test and independent sample *t*-test was used to compare the demographic and clinic characteristics differences of trial completers and non-completers at baseline.

As all variables were normally distributed and tested with Kolmogorov–Smirnov test, a two-way repeated measures analysis of variance (ANOVA) was applied to test whether the treatments were different after 6 months. Time (baseline, 3 and 6 months) was the within-group factor, groups (TC and SC) were the between-group factors and year of drug dependence was the covariate. A *post hoc* test with Bonferroni correction was used to examine which group was different if the ANOVA showed a significant interaction.

The relapse comparison was compared with independent sample *t*-test. Correlations between PSQI, SDS, and fitness at baseline were computed using Pearson's correlation analyses. Data were reported as the mean values (plus SD), and the significance level was set to *p* < 0.05.

## Results

The initial 1,181 female substance dependents were receiving treatment in SMDRC, in which 915 substance dependents were synthetic drug dependents. In addition, 82 female ATS-dependent individuals voluntarily took part in this study. A total of 80 eligible subjects were recruited and randomly assigned to the TC group (*n* = 42) and SC group (*n* = 38) (Table 1).

**Table 1:** Demography of ATS dependent (*N* = 80).

**Content**		**Tai chi (*****N*** = **42)**			**Stand care (*****N*** = **38)**		
		***N***	**Percent (%)**	**Valid percent (%)**	***N***	**Percent (%)**	**Valid percent (%)**
Education	College Level or higher	3	7	7	1	3	3
	High school or equivalent	13	31	31	6	16	16
	Middle school	23	55	55	27	71	71
	Elementary school	3	7	7	4	11	11
Occupation	Service	11	26	26	7	18	18
	Staff	2	5	5	1	3	3
	Owner	4	10	10	7	18	18
	Worker	2	5	5	2	5	5
	Farmer	1	2	2	1	3	3
	Unemployed	22	52	52	20	53	53
Marital status	Single	21	50	50	11	29	29
	Married	9	21	21	12	32	32
	Divorced/widow	12	29	29	15	39	39
Type of drug	Meth	42	46.7	100	34	57.6	89.5
	Ketamine	21	23.3	50	5	8.5	13.2
	Heroin	15	16.7	35.7	16	27.1	42.1
	Cocaine	3	3.3	7.1	1	1.7	2.6
	Ecstasy	4	4.4	9.5	1	1.7	2.6
	Marijuana	5	5.6	11.9	2	3.4	5.3
Treatment	Never	23	55	55	22	58	59
	Mental treatment	6	14	14	1	3	3
	Social assistant	1	2	2	2	5	5
	Medical treatment	12	29	29	12	32	32

**Figure 1:**
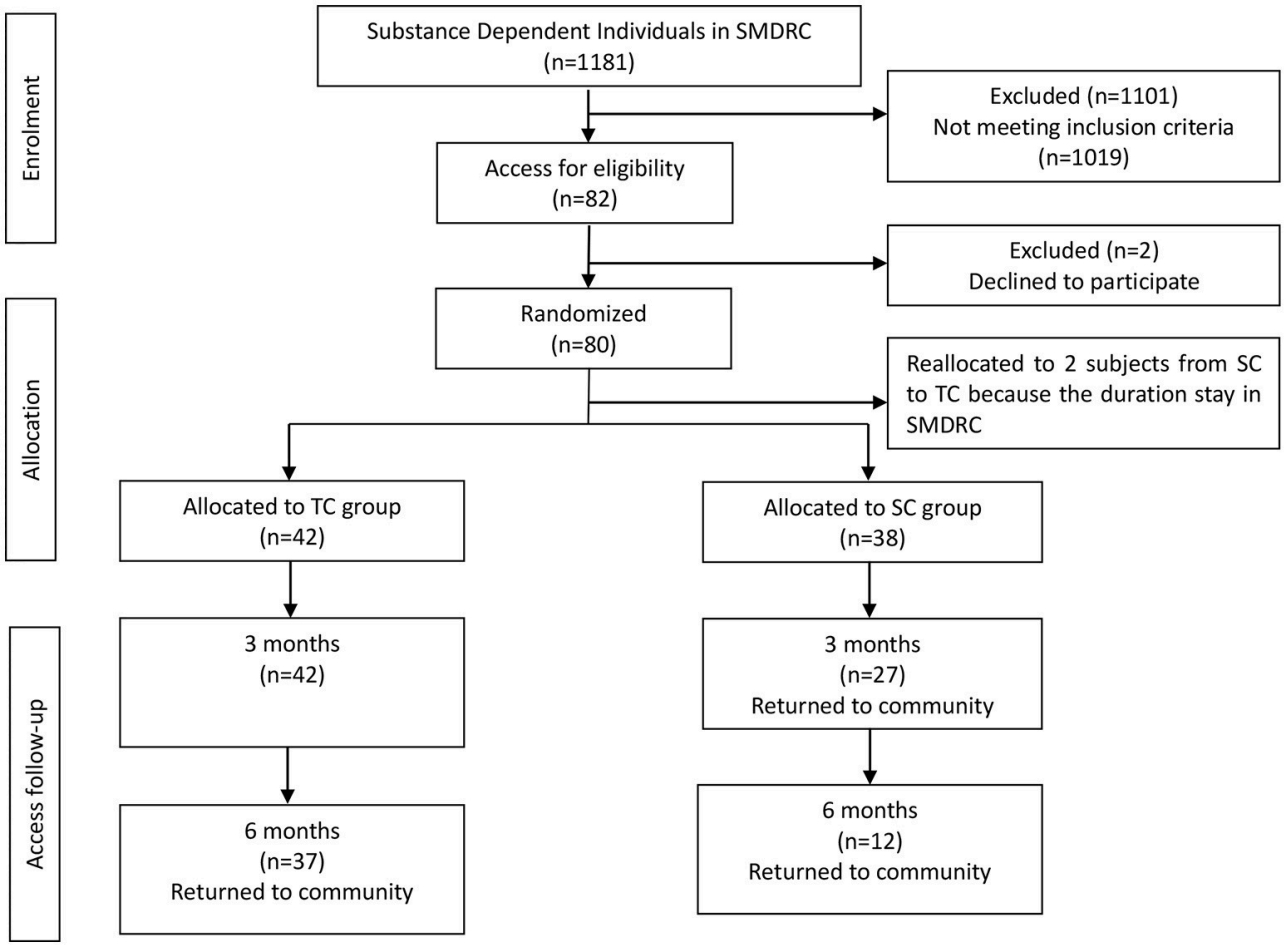
Flow diagram of the intervention progress through the phases of the two groups.

Data on subjects in the TC group were as follows: 33.74 ± 7.11 years old, 159.62 ± 5.10 cm height, 60.81 ± 6.66 kg weight, and 7 ± 4 years of ATS use. Data on subjects in the SC group were as follows: 37.76 ± 9.85 years old, 160.20 ± 5.21 cm height, 63.16 ± 7.79 kg weight, and 8 ± 6 years of ATS use. Five participants in the TC group and 26 participants in the SC group dropped out during the intervention. The reason for dropout was leaving SMDRC to the community (Figure 1).

At baseline, no statistically significant differences were observed between groups in terms of sleep quality, depression, fitness, and relapse times except for sleep duration, *t* = −2.15, *p* < 0.05; need for sleep medications, *t* = 7.66, *p* < 0.01; and PACER score, *t* = 2.48, *p* < 0.05. There was no demographic and clinic characteristics differences between trial completers and non-completers (Tables 2, 3).

**Table 2:** Baseline of PSQI, SDS, and relapse time comparison of the groups (*N* = 80).

	**Tai chi**	**Standard care**		
**Contents**	**(*****N*** = **42)**	**(*****N*** = **38)**	***T***	***p*****-value**
**Pittsburgh Sleep Quality Index**			
Subjective sleep quality, M(SD)	1.02 (0.68)	1.08 (0.78)	−0.34	0.74
Sleep latency, M (SD)	1.31 (0.72)	1.37 (0.88)		
≤15 min, n,%	5,11.9%	7,18.4%		
16–30 min, n,%	20,47.6%	13,34.2%		
31–60 min,n,%	16,38.1%	15,39.5%		
>60 min, n,%	1,2.4%	3,7.9%		
Sleep duration, M(SD)	0.24 (0.43)	0.5 (0.65)	−2.15	<0.05^*^
>7 h, n,%	32,76.2%	22,57.9%		
6–7 h, n,%	10,23.8%	13,34.2%		
5-6 h, n,%	0	3,7.9%		
Habitual sleep efficiency, M(SD)	92.50 (6.59)	88.50 (11.45)	1.94	0.06
≥85%, n,%	35,83.4%	27,70.9%		
75–84%, n,%	7,16.7%	7,18.4%		
65–74%, n,%	0	1,2.6%		
<65%, n,%	0	3,7.8%		
Sleep disturbances, M(SD)	1.21 (0.47)	1.34 (0.58)	−1.08	0.28
Need for sleep medications, M(SD)	0.88 (0.67)	0.03 (0.16)	7.66	<0.001^**^
Daytime dysfunction	1.10 (0.91)	1.11 (1.09)	−0.05	0.96
PSQI score	5.07 (2.44)	5.89 (3.25)	−1.29	0.2
Self-rated Depression Scale	48.55 (8.25)	49.95 (9.29)	−0.71	0.48
History of relapse (n)	1.81 (1.04)	2.03 (1.17)	0.77	0.38

Data presented as mean (SD);

* p < 0.05;

** *p < 0.01*.

**Table 3:** Baseline of fitness comparison of the groups (*N* = 80).

	**Tai chi**	**Standard care**		
**Contents**	**(*****N*** = **42)**	**(*****N*** = **38)**	***T***	***p*****-value**
Mass (kg)	60.81 (6.66)	63.16 (7.79)	−1.4	0.15
Body fat (%)	31.24 (2.48)	32.50 (4.28)	−1.6	0.12
Systolic (mmHg)	121.40 (14.71)	119.61 (11.69)	0.6	0.55
Diastolic (mmHg)	78.40 (10.77)	79.50 (10.50)	−0.46	0.65
Pulse (bmp)	85.45 (9.64)	80.97 (12.26)	1.83	0.07
Vital capacity (ml)	2699.07 (727.14)	2571.24 (678.81)	0.81	0.42
Hand grip (R) (kgf)	24.57 (5.30)	24.07 (5.51)	0.41	0.68
Sit-and-reach (cm)	11.60 (6.66)	8.61 (7.95)	1.83	0.07
One-leg stand with eye closed (s)	26.93 (25.55)	31.65 (30.74)	−0.75	0.46
PACER (laps)	20.93 (7.47)	16.95 (6.68)	2.48	< 0.05^*^

Data presented as mean (SD);

* *p < 0.05*.

### Sleep quality (PSQI)

Significant differences were found regarding the scores of sleep duration [*F*_(2, 92)_ = 9.86, *p* < 0.001, η^2^ = 0.18] by time × group interaction after 6 months. The *post hoc* test further revealed a significantly long sleep duration in the TC group [*F*_(1, 46)_ = 23.75, *p* < 0.001]. Need for sleep medications [*F*_(2, 92)_ = 36.44, *p* < 0.001, η^2^ = 0.44] and daytime dysfunction [*F*_(2, 92)_ = 5.15, *p* = 0.01, η^2^ = 0.10] were found to be significantly different by time × group interaction after 6 months. Although the scores of PSQI showed no significant difference between the two groups by time × group interaction after 6 months, the *post hoc* test revealed that PSQI score [*F*_(1, 46)_ = 5.19, *p* = 0.027, η^2^ = 0.10] and habitual sleep efficiency [*F*_(1, 46)_ = 8.05, *p* = 0.007, η^2^ = 0.15] in the TC group significantly decreased statistically compared with those in the SC group. The PSQI score with the repeated measure by group × year of drug dependence revealed significant differences, *F*_(2, 92)_ = 5.21, *p* = 0.007.

Between-group results showed that sleep duration [*F*_(1, 46)_ = 23.75, *p* < 0.001], habitual sleep efficiency [*F*_(1, 46)_ = 8.05, *p* = 0.007], need for sleep medications [*F*_(1, 46)_ = 35.4, *p* < 0.001], and PSQI score [*F*_(1, 46)_ = 5.19, *p* = 0.027] were significantly different (Table 4).

**Table 4:** Comparison of two groups at baseline, 3 and 6 months by ANOVA repeat measures (*N* = 49).

	**Tai chi (*****N*** = **37)**			**Standard care (*****N*** = **12)**					
	**Baseline**	**3 month**	**6 month**	**Baseline**	**3 month**	**6 month**	**Within-group**	**Between-group**	**Group × time**
**PITTSBURGH SLEEP QUALITY INDEX**									
Subjective sleep quality	1.03 (0.69)	0.73 (0.65)	0.70 (0.70)	0.92 (0.79)	0.92 (0.99)	0.67 (0.78)	0.41	0	0.91
Sleep latency	0.84 (0.69)	0.81 (0.84)	0.70 (0.62)	1.00 (0.95)	1.25 (1.14)	0.83 (0.94)	3.26^*^	1.33	0.85
Sleep duration	0.24 (0.44)	0.24 (0.49)	0.11 (0.31)	0.58 (0.67)	0.75 (0.75)	1.17 (0.58)	0.51	23.75^**^	9.86^**^
Habitual sleep efficiency (%)	92.5 (6.81)	91.86 (9.05)	93.16 (5.04)	87.92 (11.13)	84.17 (14.34)	86.33 (13.70)	0.51	8.05^**^	0.43
Sleep disturbances	1.24 (0.43)	1.11 (0.39)	1.03 (0.29)	1.42 (0.51)	1.17 (0.39)	1.08 (0.29)	2.43	0.89	0.48
Need for sleep medications	1.00 (0.62)	0.00 (0.00)	0.03 (0.16)	0.00 (0.00)	0.00 (0.00)	0.00 (0.00)	3.45^*^	35.4^**^	36.43^**^
Daytime dysfunction	1.05 (0.91)	0.46 (0.73)	0.41 (0.60)	1.08 (0.90)	0.83 (0.72)	0.08 (0.29)	4.89^*^	0.01	5.15^**^
PSQI score	5.03 (2.55)	3.78 (2.76)	3.32 (1.92)	6.08 (3.06)	6.08 (3.42)	4.50 (2.51)	2.85	5.19^*^	2.11
Self-rated Depression Scale	48.19 (8.12)	48.86 (9.66)	46.14 (10.67)	48.25 (9.08)	52.00 (7.59)	49.25 (8.31)	4.10^*^	0.62	0.84
**FITNESS OUTCOME**									
Mass (kg)	61.57 (5.51)	60.27 (5.77)	61.51 (5.77)	64.33 (8.63)	62.83 (9.33)	61.51 (5.77)	2.42	1.53	0.4
Body fat (%)	31.33 (2.49)	30.86 (2.40)	32.61 (2.87)	32.93 (4.86)	31.97 (4.60)	32.97 (5.11)	6.01^**^	1.05	1.88
Systolic (mmHg)	121.59 (15.29)	109.14 (10.63)	110.46 (9.10)	119.67 (13.56)	111.83 (10.43)	117.75 (9.76)	6.01^**^	0.75	2.27
Diastolic (mmHg)	78.46 (11.13)	68.16 (8.20)	72.68 (7.24)	82.00 (12.29)	70.75 (10.76)	77.58 (10.67)	7.28^**^	2.07	0.23
Pulse (bmp)	85.65 (9.57)	70.84 (7.25)	72.78 (8.11)	81.17 (13.54)	72.83 (7.70)	75.33 (13.97)	14.69^**^	0	3.08*
Vital capacity (ml)	2641.32 (725.48)	2288.38 (580.20)	2207.32 (476.78)	2618.17 (697.63)	2169.83 (656.14)	2127.00 (399.31)	5.20^**^	0.24	0.09
Hand grip (R) (kgf)	24.59 (5.42)	25.76 (4.17)	33.03 (5.73)	24.27 (5.52)	24.24 (5.73)	32.92 (4.31)	84.41^**^	0.16	0.87
Sit-and-reach (cm)	10.86 (6.61)	13.82 (5.48)	10.65 (5.16)	8.09 (7.71)	14.35 (13.83)	8.82 (5.80)	3.48^*^	0.51	1.14
One-leg stand with eye closed (s)	28.75 (26.61)	36.53 (27.93)	45.36 (37.46)	29.36 (23.07)	21.30 (13.01)	27.97 (13.46)	0.7	1.96	2.02
PACER (laps)	20.51 (7.35)	14.14 (5.41)	17.41 (8.24)	16.92 (8.39)	13.33 (5.73)	12.42 (3.73)	1.95	2.97	1.49

Data presented as mean (SD);

* p < 0.05;

** *p < 0.01*.

### SDS

Although no significant difference was observed between the two groups, the score of SDS decreased in the TC group, whereas that in the SC group was the same after 6 months (Table 4).

### Fitness outcome

Our findings showed that the pulse rate had significantly decreased in the TC group compared with the SC group [*F*_(2, 92)_ = 3.32, *p* = 0.04] after 6 months by time × group interaction.

Between-group test by repeated measures ANOVA revealed significant differences between groups in terms of body fat [*F*_(1, 46)_ = 4.49, *p* = 0.04] and running laps of PACER [*F*_(1, 46)_ = 9.83, *p* = 0.003]. Compared with the baseline, the results of PACER in both groups had decreased. A significant difference was observed in the between-group test by ANOVA with years of drug dependence, *F*_(1, 46)_ = 9.83, *p* < 0.003 (Table 4).

### Relapse

Independent *t*-test revealed a significant difference between the two groups in terms of relapse (*t* = 3.37, *p* = 0.006). The relapse in the TC group was 9.5% and that in the SC group was 26.3% because ATS-dependent individuals had left SMDRC. The relapse investigation further demonstrated that the duration of ATS cessation among relapse individuals was significantly different (*t* = 4.94, *p* < 0.01). The cessation durations of ATS-dependent individuals from the last relapse to be found this time to relapse were 1,209 and 880 days in the TC and SC groups, respectively (Table 5).

**Table 5:** Follow-up Investigation of Relapse (*N* = 80).

**Contents**	**Tai chi**	**Standard care**	***T***	***p*-value**
Relapse (N)	4 (9.5%)	10 (26.3%)	3.37	0.006^**^
Duration of ATS Cessation (Days)	1209 (79)	880 (121)	4.95	0.00^**^
Numbers of Cessation (N)	38 (90.5%)	28 (73.7%)	3.9	0.048^*^

Data presented as mean (SD);

* p < 0.05;

** *p < 0.01*.

### Associated factors among ATS-dependent individuals

The Pearson correlation test revealed that several significant results were associated with baseline outcomes. The correlation between PACER and the score of SDS was *r* = 0.30, *p* = 0.008. The correlation between PACER and the years of illicit drug use was *r* = −0.33, *p* = 0.003. The correlation between total score of PSQI and the years of illicit drug use was *r* = 0.27, *p* = 0.016.

## Discussion

To our knowledge, this paper is the first to discuss the effect of TC intervention on female ATS-dependent individuals. Our study found that ATS-dependent individuals in the TC group had a better score of PSQI and SDS and some positive changes in terms of fitness compared with the SC group. A follow-up relapse investigation demonstrated that participants in the TC group had less relapse.

### Sleep quality and mood

The main outcome of PSQI showed that ATS-dependent individuals in the TC group had a better PSQI score than those in the SC group. The duration of sleep and daytime dysfunction were found to have significantly improved in the TC group. Furthermore, ATS-dependent individuals in the TC group had shorter sleep latency than that in the SC group after 6 months of treatment. At baseline, the sleep duration and need for sleep medication had significant differences as the result of the high percentage of participants in the TC group that used sleep medication. With the intervention, participants in the TC group that needed sleep medication decreased from 71.5% (30) to 2.7% (1) after 6 months.

To date, a cross-sectional study on the sleeping problem in Chinese illicit drug-dependent subjects has been conducted (Tang et al., 2015). The study investigated a total of 2,178 illicit drug users and 2,236 non-drug users in Changsha, China. The PSQI score in our study at baseline was slightly lower than that in the report (7.97 ± 4.39). The reason for this difference is that subjects were collected following a 10-day detoxification treatment in the earlier study, while in our study, all subjects had experienced detoxification and rehabilitation for at least 6 months at SMDRC. Interestingly, the PSQI score in the TC group after 6 months of intervention in our study was lower than that of non-drug users reported by Tang et al., but the PSQI score in the SC group was close to what that study displayed (4.20 ± 2.468).

While practicing TC, the mind should be calm. Integrating movements and the spirit is required, and breathing shall be harmonious with movements. The breathing pattern may alter the functioning of the autonomic nervous system. Various breathing practices are believed to be beneficial to release emotion, calm the mind, or enhance physical power, all of which may be regarded as potential mediators of improved sleep quality (Payne and Crane-Godreau, 2013). Evidences indicated that TC can effectively improve sleep quality not only for normal older adults but also for older adults with cognitive impairment and elderly Chinese women with knee OA (Li et al., 2004; Chan et al., 2016; Lü et al., 2017). A systematic follow-up review reported positive effects of TC on the executive function of cognitively healthy adults compared with no intervention, other active interventions and exercise (Solloway et al., 2016). The rationale of TC ameliorating sleep and mood disturbance is that moderate rhythmic movement may increase the parasympathetic tone, whereas intense exertion causes further sympathetic activation. TC is a moderate-intensity exercise. The smooth rhythmic motions of TC are usually experienced as relatively pleasurable mood (Pa et al., 2014; Kim et al., 2016). Our study demonstrated that TC may be a potential physical activity to escalate sleep quality and fitness for female ATS dependents.

Numerous studies have shown high prevalence of sleep disturbance among illicit drug dependents (Mahfoud et al., 2009; Brower and Perron, 2010; Liao et al., 2011; Mahoney et al., 2014; Tang et al., 2015). Similarly, our findings showed that the PSQI score was associated with the duration of illicit drug use. In addition, aerobic capacity tested by PACER was associated with illicit drug use and depression.

In our study, the scores of self-reported depression had slightly decreased in the TC group, but no changes in the SC group were found. Thus, TC has therapeutic value for the sleep quality and depression of ATS dependents. Disturbed sleep is an important predictor of relapse. However, we compared the PSQI score with the non-relapse and relapse ATS-dependent individuals in our study. We found no significant differences. A noteworthy comparison between non-relapse and relapse individuals in our study was the balance outcome at the baseline. Findings showed that the re-relapse ATS-dependent individuals had shorter balance time (19 s) than ATS-dependent individuals who have not been found to relapse after they left SMDRC (they had a longer balance time: 31 s).

### Fitness changes

The fitness test results showed an improvement in the balance control but no significant differences between groups. Numerous studies have suggested that TC can efficiently improve the functional balance (Province et al., 1995; Campbell et al., 1997). The TC movements in the intervention included “golden rooster stands on one leg” (Jing Ji Du Li). The TC instructors observed that the participants in the TC group swayed their trunks while performing Jing Ji Du Li and when they were shifting their weight from one leg to another in the first 2 weeks. This outcome may be considered as a result from long-term ATS abuse, which negatively affected the proprioception of the participants, and the fact that they were unfamiliar with the TC movements before the intervention. The balance mechanism developed by the repeated positioning of the body and limb joints in specific spatial positions through TC can induce plastic changes in the cortex. The repetitive, afferent inputs from the cutaneous receptors in the skin and limb proprioceptors, including muscle and tendon spindles, can modify the cortical maps of the body over time (Tsang and Hui-Chan, 2003).

Blood pressure also decreased in the TC group but slightly changed in the SC group. The systolic pressure had decreased in the TC group by 11 mmHg, with no change in the SC group. The post-intervention changes in the systolic and diastolic pressures are consistent with the findings from earlier studies that reported a decrease in blood pressure after TC practice (Chen et al., 2012) (Wolf et al., 2003; Zhu et al., 2016). These changes were not associated with the antihypertensive use by ATS-dependent individuals. The record of antihypertensives indicated that four ATS dependents in each group used a Zheng Ju antihypertensive, which is a Chinese herb pill. Although the pulse decreased in both groups, results displayed that the ATS dependents' pulse in the TC group decreased significantly after 6 months of TC practice. Yoga, TC, meditation and other relaxation therapies have been reported to reduce sympathetic activity, decrease sympathoadrenal reactivity and enhance parasympathetic output, which may, in turn, reduce the prevalence and severity of vasomotor disturbances and sleep impairment (Innes et al., 2010).

Notably, the PACER score, which represents the aerobic capacity of ATS dependents, decreased in both groups. However, although the aerobic capacity in the TC group decreased after 3 months, it had increased after 6 months. By contrast, the aerobic capacity in the SC group decreased rapidly. This result indicated that TC can slow down the decreasing trend of the aerobic capacity of ATS dependents. Furthermore, female ATS dependents in SMDRC are sedentary patients. Effective exercises shall be widely applied in mandatory detoxification and rehabilitation centers in China.

In accordance with other studies in China, after detoxification, the relapse rate for heroin abusers within the first month is 54.57%. The relapse rate within the first to third months is 31.76%, whereas it is 93.31% within the first 6 months and 96.68% within the first year. Drug addicts often fall into the vicious cycle of “drug-taking–detoxification–relapse–effort–quit” (Jia et al., 2015). The relapse investigation was conducted with relapse data examination instead of PSQI, SDS and fitness measurement. Our study indicated that TC has positive effects on sleep quality, depression and fitness and participants in the TC group had low relapse percentage. The long-term study demonstrates that TC may be a cheap and potential supplementary treatment for ATS-dependent individuals. TC may also be considered as an alternative exercise to escalate abstinence for female ATS-dependent individuals.

## Limitation

This study has a number of limitations. First, although the participants were randomized into the TC and SC groups, the groups had to be slightly modified due to the reallocation of their residence in the SMDRC. Second, the number of dropouts in the SC group was higher than that in the TC group. The reason for dropout was not associated with the different interventions or the performance of instructors in the two groups. The findings must be interpreted with caution as the high attrition rate recorded in the SC group at 3 and 6 months can affect the sample's representativeness. Future study should consider participants' expected number of treatment days in mandatory and detoxification centers before setting up an intervention. Third, although the relapse assessment was from the data of NSSDA and SSDC, it is possible some relapsing individuals may not have been identified by the surveillance system. Nevertheless, this was the first study to report the longitudinal effect of TC exercise intervention. Forth, the intensity of our interventions was fixed, future studies should consider the effects of exercise duration.

## Ethics statement

This study was carried out in accordance with the recommendations of Policies and Procedures for Projects that Involved Human Subjects, Research Ethic Committee of Shanghai University of Sport with written informed consent from all subjects. All subjects gave written informed consent in accordance with the Declaration of Helsinki. The protocol was approved by the Research Ethic Committee of Shanghai University of Sport.

## Author contributions

DZ conceived the trial, designed the exercise intervention, wrote and revised the manuscript. GD acquired partial financial support for the research project, and partially worked on the discussion part. DX coordinated the research project and collected the data of ATS dependents' relapse. XX revised the English grammar and abstract. JG conducted the exercise intervention and data collection. WZ contributed the idea of this trial and partially discussed the data. XJ contributed to the trial design and data collection. MT contributed to the trial design and revised the manuscript.

### Conflict of interest statement

The authors declare that the research was conducted in the absence of any commercial or financial relationships that could be construed as a potential conflict of interest.
